# Slow Cortical Potentials and Amplification—Part I: N1-P2 Measures

**DOI:** 10.1155/2012/921513

**Published:** 2012-10-18

**Authors:** Susan Marynewich, Lorienne M. Jenstad, David R. Stapells

**Affiliations:** School of Audiology and Speech Sciences, The University of British Columbia, 2177 Wesbrook Mall, Room 443, Vancouver, BC, Canada V6T 1Z3

## Abstract

Slow cortical potentials (SCPs) are currently of great interest in the hearing aid fitting process for infants; however, there is conflicting evidence in the literature concerning the use of SCPs for this purpose. The current study investigated SCP amplitudes and latencies in young normal-hearing listeners in response to a 60 ms duration tonal stimulus (1000 Hz) presented at three intensities (30, 50, and 70 dB SPL) in aided and unaided conditions using three hearing aids (Analog, DigitalA, and DigitalB) with two gain settings (20 and 40 dB). Results showed that SCP amplitudes were smaller for the digital hearing aids compared with the analog hearing aid, and none of the hearing aids resulted in a reliable increase in response amplitude relative to the unaided across conditions. SCP latencies in analog conditions were not significantly different from latencies in the unaided conditions; however, both digital hearing aids resulted in significantly delayed SCP latencies. The results of the current study (as well as several previous studies) indicate that the SCP may not accurately reflect the amplified stimulus expected from the prescribed hearing aids. Thus, “aided-SCP” results must be interpreted with caution, and more research is required concerning possible clinical use of this technique.

## 1. Introduction

With the advent of Universal Newborn Hearing Screening programs, it has become increasingly common for infants to be fitted with hearing aids by six months of age, which requires that reliable methods are in place for fitting hearing aids in this young population [1–4]. There are two general categories of test procedures used in the hearing aid fitting process: (i) behavioural measures, which require active participation by the patient (e.g., pure-tone thresholds, speech testing, and self-report questionnaires), and (ii) objective measures, which require no subjective responses from the patient (e.g., real-ear electroacoustic and evoked potential measures, especially of threshold). In infants, behavioural measures are much more limited: behavioural thresholds are not usually available before the age of 6 months (and often later), speech testing is unavailable, and subjective questionnaires are limited to caregiver observation of behaviours [2]. Thus, there is greater reliance on objective measures such as auditory evoked potentials (AEPs) [1, 5, 6] rather than the subjective measures possible with most adults [2, 7, 8]. AEPs have been considered for use within the hearing aid fitting process for at least two different purposes: (i) to determine whether aided AEPs can be obtained at input levels where unaided AEPs were absent and/or (ii) to determine whether changes in hearing aid settings (e.g., gain) and/or stimuli can be measured using AEPs. Several AEPs have been assessed as potential objective measures, including the auditory brainstem response (ABR), the auditory steady-state response (ASSR), and the slow cortical potential (SCP).

Previous ABR research revealed that the click and brief-tone stimuli required for ABR testing are too short to activate the compression processing and steady-state response of the hearing aid; thus, the ABR has been deemed not to be suitable for assessment of responses to hearing-aid-processed stimuli [9–11]. In light of these findings, subsequent researchers have considered the 80-Hz ASSR as a solution to the stimulus problem (e.g., [12–14]). For the purpose of hearing aid measures, the potential advantage of ASSR stimuli over clicks and brief tones is that they are continuous, steady-state stimuli with low crest factors, which allow the hearing aid to settle into its steady state [13]. However, recent research suggests that although continuous stimuli are used to elicit the 80-Hz ASSR, the responses likely reflect only the initial portion of the stimulus, much like the ABR [15]. Thus, the 80-Hz ASSR may be subject to the same limitations as the ABR [16] and, like the ABR, shows poorer frequency specificity than indicated by the acoustics [17, 18]. Both the ABR and 80-Hz ASSR share the limitation of being brainstem responses and, as a result, do not give any indication of higher-level (cortical) processing.

Slow cortical potentials have an advantage over brainstem AEPs because they originate in the auditory cortex [19–21] and can be elicited by a variety of signals, including tonal stimuli longer than those used for the ABR as well as speech stimuli (for reviews, see: [22, 23]). As a result, the SCP is now the AEP of greatest interest for use with hearing aids [24–30]. For example, several studies using subjects with various degrees, types, and configurations of hearing loss and their personal hearing aids have shown that SCPs show some promise in the hearing aid fitting process using a variety of stimuli, including tonal and complex ones [27–29, 31–33]. Findings such as these have led a group of researchers at the National Acoustic Laboratories to develop a new device currently being marketed as a hearing aid validation tool for infants and children [34, 35].

Despite the current upsurge of interest in the use of cortical SCPs with hearing aids in infants and children, there are several recent studies which indicate that concerns exist regarding the use of SCPs for this purpose. For example, some recent studies on SCPs in subjects with normal hearing using speech and tonal stimuli have found that the addition of hearing aid gain does not lead to the expected increase in N1-P2 amplitude that occurs in response to higher stimulus levels, such that there were either no significant differences between unaided and aided N1-P2 amplitudes [25, 30] or *smaller* aided N1-P2 amplitudes compared to unaided [24]. Billings and colleagues [24, 36] have examined the effect of SNR (i.e., stimulus amplitude to background noise ratio) on aided and unaided SCPs and, as a result, have concluded that the lack of an amplification effect on the SCP seen in their earlier work [25, 30] was due, at least in part, to the SNR being similar across aided and unaided conditions (i.e., in addition to amplifying the stimulus, the hearing aid also introduced higher noise levels).

The stimuli used by Billings and colleagues [24, 25, 30, 36] were atypical for SCP stimuli in that they had a more rapid rise time (7.5 ms) than is optimal for eliciting the SCP, and they were much longer in duration (756 ms) than is known to be reflected by the SCP. Research has shown that N1 shows little effect of stimulus changes beyond the first 20–40 ms [37–41]. Also, rise times between 20 and 30 ms result in the largest N1 amplitudes with either no further change, or decrease, in amplitude with longer rise times [40, 41]. Perhaps different results may have been obtained using stimuli with more typical rise/fall times and overall durations, such as 20 ms rise time and 60 ms total duration [22, 23, 42–44], although a recent study by Easwar and colleagues [45] suggests little difference in SCPs to hearing-aid-processed stimuli with 7.5 ms versus 20 ms rise times.

Due to conflicting evidence regarding the use of SCPs for hearing aid measurements, the primary aims of the current study were: (i) to determine the effects of hearing aid gain on the SCP and, specifically, whether different hearing aid gains are accurately reflected by the SCP, and (ii) to assess whether results are similar for different hearing aids set with the same gain characteristics. Of particular interest was whether there would be a difference between unaided and aided response amplitudes and latencies when hearing aids were set for 20 or 40 dB of gain and whether unaided and aided response amplitudes and latencies would be comparable for equivalent nominal output levels.

## 2. Methods

### 2.1. Subjects

Thirteen normal-hearing subjects participated in this study (mean age: 25 ± 5.5 years; 5 females). Subjects were briefed on the study procedures and provided informed written consent prior to participating. All subjects were screened for normal hearing by behavioural audiometry and for normal middle/outer ear function by immittance audiometry. Normal hearing was defined by pure-tone behavioural thresholds equal to or better than 15 dB HL from 500 to 4000 Hz and equal or better than 20 dB HL at 250 and 8000 Hz [46]. Normal tympanograms were defined by a single-peak static admittance between ±50 daPa in response to a 226-Hz probe tone. Subjects were excluded if: (i) SCPs were absent in any of the unaided conditions, or (ii) SCPs were absent in three or more aided conditions. Two subjects (not included in the above numbers) were excluded from the study on the basis of these criteria.

### 2.2. Recording

One electroencephalogram (EEG) channel was recorded from electrodes placed at Cz and M1. A second channel to monitor vertical eye movements and eye blinks (EOG) was recorded from electrodes over the left supraorbital ridge of the frontal bone and over the zygomatic bone under the left eye. A fifth electrode on the nape of the neck served as ground. Electrode impedances were maintained below 5000 Ohms. Recordings were made using Neuroscan Synamps 2 and Scan 4.3 software. The EEG and EOG channels were amplified, filtered (1–30 Hz), and digitized (5000 Hz), using a 700 ms analysis time (including a 100 ms prestimulus baseline). Single-trial epochs were saved for offline processing, including baseline correction across the total sweep duration, artifact rejection (±100 *μ*V in any of the channels, and ±75 *μ*V in the EOG channel), and averaging. The stimulus was presented in each test condition until at least 200 accepted trials were obtained. Epochs were averaged separately for each condition, and average data were divided into odd and even trials to serve as replications (thereby ensuring any changes in subject state or noisiness were equivalent across replications). Averages were baseline-corrected using the prestimulus interval.

### 2.3. Hearing Aids

The same three behind-the-ear stock hearing aids, coupled with Comply snap tip 9-mm foam earmolds inserted to be flush with the ear canal entrance, were used for each participant: (i) Oticon E27 (Analog), (ii) Phonak Savia 211 dSZ (DigitalA), and (iii) Siemens Acuris S (DigitalB). Two digital hearing aids were selected because digital signal processing is currently the most commonly used technology; therefore, SCP results using digital hearing aids are the most clinically relevant. An analog hearing aid was selected to account for possible discrepancies between results for digital hearing aids and those in the published literature which primarily used analog hearing aids [24, 25, 28, 30, 31, 33].

The digital hearing aids were programmed using NOAH 3 software and the NOAHlink programming assistant. Two gain settings (20 and 40 dB) were required; therefore, for each subject, two programs were created and the gain settings were verified by real-ear insertion gain (REIG) measures. The 20 dB gain setting was chosen to approximate the hearing aid setting used by Billings et al. [25], and the 40 dB gain setting was added to assess whether additional gain would result in a significant difference between unaided and aided SCPs. Both programs were set with a 1 : 1 compression ratio and were verified for linear processing using input/output coupler measures. All additional hearing aid features such as digital noise reduction and feedback management were disabled. Maximum output was set to the highest level. Settings for the digital instruments were saved in the NOAH 3 software for each subject so that hearing aid programs could be recalled in follow-up sessions. Gain settings for the analog hearing aid were achieved by setting the volume control to one (minimum) and turning the dB SPL trim-pot until the REIG was 20 dB at 1000 Hz. To achieve the 40 dB gain setting, the volume control wheel was turned up until REIG equalled 40 dB at 1000 Hz. The volume control wheel was then marked for that setting. Unlike the digital hearing aids, gain settings for the Analog hearing aid had to be re-measured in follow-up sessions.

REIG was determined for each individual participant using the Fonix 7000 real-ear system. REIG was chosen because it equals the difference between unaided and aided responses in the ear canal, which most closely approximates the comparisons made in these studies (i.e., SCPs and acoustic measures were conducted in both unaided and aided conditions). A small probe tube (3 mm in diameter) was placed in the ear canal of the participant within 5 mm of the eardrum (verified by otoscopy). The tube was then marked at the tragal notch to ensure identical probe-tube placement across hearing aids. The gain control setting at 1000 Hz was adjusted until the appropriate REIG level was achieved for each program; other frequencies were set to provide the least amount of gain possible.

A swept pure tone with constant input level across the frequency range was used to measure REIG. A pure-tone stimulus was used as this is the same type of stimulus (i.e., tonal, rather than speech-like) used to elicit the SCPs. A 50-dB SPL input level was used to program both 20- and 40 dB REIG settings, and a 70-dB SPL input level was used to verify the 20 dB REIG settings; these were the same input levels and gains used in the SCP testing. For all participants, all hearing aids matched target gain within 1 dB for all input levels used in SCP testing, except 30 dB SPL, which could not be verified as it was below the levels available in the hearing aid test system. These measures provided further verification that all three hearing aids were providing linear processing and that all measures were below the maximum output of the hearing aid.

Electroacoustic measures of processing delay were conducted on the Fonix 7000 test system. The delays were 0.4 ms for the Analog aid, 6.8 ms for DigitalA, and 2.3 ms for DigitalB.

### 2.4. SCP Stimuli

The stimulus used was a 1000-Hz tonal stimulus of a 60 ms total duration (including a 20 ms rise/fall time). A 20 ms rise/fall time was chosen because it is suitable for generating a large N1 response, and the 60 ms total duration was chosen because it has been shown that stimuli of longer duration do not result in increased N1 amplitudes [37–41]. Stimuli were presented with offset-to-onset interstimulus intervals (ISIs) of 940 ms. Stimuli generated by Neuroscan's Stim 2 software were further amplified by a Wavetek Rockland 852 filter (providing 20 dB of amplification below 3000 Hz), and routed through a Tucker Davis Technologies (TDT) PA5 attenuator and HB7 headphone driver, and finally to a speaker in the sound field placed at 1.5 meters from the subject at 0° azimuth. The stimulus output at 80 dB SPL was calibrated with a Larson Davis sound level meter by measuring the level of a longer-duration 1000-Hz tone (2-s duration and 20 ms rise/fall time; equal in peak-to-peak amplitude to the 60 ms 1000-Hz stimulus) at the head height of the subject, 1.5 m from the speaker. Stimuli were presented at three intensities (30, 50, and 70 dB SPL). A maximum stimulus intensity of 70 dB SPL was chosen to limit the maximum signal to 90 dB SPL, after hearing aid gain. For the 40 dB gain condition, the maximum stimulus intensity was 50 dB SPL, again to limit the maximum signal to 90 dB SPL. For all conditions, the subject's left ear was plugged with a deeply seated foam plug in order to reduce any contributions of responses resulting from stimulation to the non-test ear.

### 2.5. Procedure

Participation in this study involved two sessions on separate days, each 2 -3 hours in length (i.e., 4–6 hours total). Subjects were screened for normal hearing and for normal outer- and middle-ear function. Immittance audiometry was also conducted in the second test session to ensure no changes across test sessions.

Following hearing aid programming, all testing was conducted in a double-walled sound-attenuating booth. Average octave band noise levels in the sound-attenuated booth at 500, 1000, 2000, and 4000 Hz were 12, 10, 10, and 12 dB SPL, respectively. There were 18 test conditions, and the presentation order for each subject was randomly assigned prior to the test dates. During testing, participants were asked to sit as still as possible while watching a movie of their choice in closed captioning and no sound. Subjects sat in a reclining chair set in the upright position so that each participant was seated with their head above the chair back. This position was chosen because (i) calibration measures showed that it was the “flattest” spot in the soundfield, making it appropriate for the substitution method of calibration employed in these measures, and (ii) it was easy to monitor via closed-circuit television whether the participant's head position had moved substantially out of the calibrated spot in the soundfield.

### 2.6. Data Analysis

SCP measures of interest were N1-P2 peak-to-peak amplitude and N1 latency. N1 peak amplitude measures were determined at the largest negativity occurring before 200 ms, and P2 peak amplitude measures were determined at the largest positivity within 100 ms of N1; N1 latency was measured at the centre of the peak. In cases of multi-peaked waveforms, amplitude measures were taken at the largest amplitude and N1 latency was taken as the midpoint of the two negative peaks [28, 47–50]. For a response to be “present”, N1 was required to be replicable across odd and even average waveforms. If responses were “absent”, a value of 0 *μ*V was substituted as a reasonable estimate of amplitude (e.g., [28, 47–50]). Latencies were not estimated for no-response results. Due to absent responses for 5 out of 13 subjects in some of the 30 dB SPL conditions, latency results for this input level were excluded from statistical analyses.

#### 2.6.1. Statistical Analysis

For amplitude measures, four repeated-measures analyses of variance (ANOVA) were conducted: (i) to measure the effect of the 20 dB gain setting, a two-way repeated-measures ANOVA was conducted comparing four levels of hearing aid type (unaided, Analog, DigitalA, and DigitalB) and three input levels (30, 50, and 70 dB SPL), (ii) to measure the effect of the 40 dB gain setting, a two-way repeated-measures ANOVA was conducted comparing four levels of hearing aid type (unaided, Analog, DigitalA, and DigitalB) and two input levels (30 and 50 dB SPL), (iii) to compare 20 and 40 dB gain settings, a three-way repeated-measures ANOVA was conducted comparing two levels of gain (20 and 40 dB), three levels of hearing aid type (Analog, DigitalA, and DigitalB), and two input levels (30 and 50 dB SPL), and (iv) to compare results for a 70 dB SPL “nominal output” (i.e., input intensity plus gain), a one-way repeated-measures ANOVA with seven “conditions” was conducted (i.e., unaided 70-dB SPL input level condition, 50 dB SPL input level plus 20 dB gain condition for the Analog, DigitalA, and DigitalB hearing aids, and 30 dB SPL input level plus 40 dB gain condition for the Analog, DigitalA, and DigitalB hearing aids).

For latency measures, the following three repeated-measures ANOVAs were conducted: (i) to measure the effect of the 20 dB gain setting, a two-way repeated-measures ANOVA was conducted comparing four levels of hearing aid type (unaided, Analog, DigitalA, and DigitalB) and two input levels (50 and 70 dB SPL), (ii) to measure the effect of the 40 dB gain setting, a one-way repeated-measures ANOVA was conducted (because the 30 dB SPL input level was excluded) comparing four levels of hearing aid type (unaided, Analog, DigitalA, and DigitalB) at one input level (50 dB SPL), and (iii) to make comparisons between the 20 and 40 dB gain settings, a one-way repeated-measures ANOVA was conducted comparing three levels of hearing aid type (Analog, DigitalA, and DigitalB) at 50 dB SPL. Analysis of “nominal output” was not conducted for latency measures due to the absence of latency results for the 30 dB SPL input level and thus the exclusion of the 40 dB gain condition.

For all analyses, the main effects and interactions were considered significant if *P* < 0.05. Huyn-Feldt epsilon (*ε*) correction factors for repeated measures were applied to the degrees of freedom and are reported where appropriate. Neuman-Keuls post hoc analyses were performed for significant main effects or interactions. Post hoc analyses were considered statistically significant if *P* < 0.05.

## 3. Results

**Figure 1:**
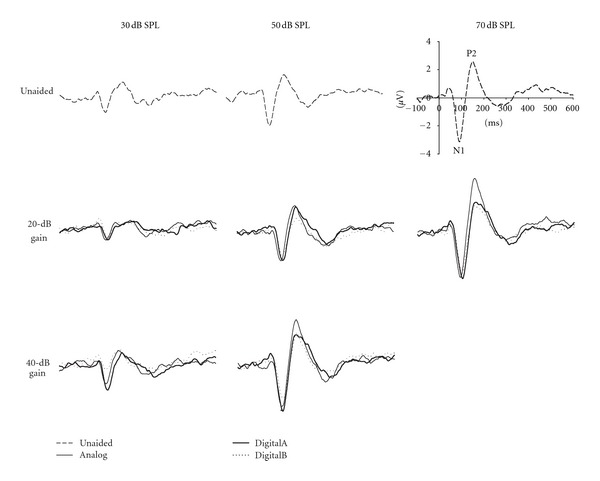
Grand mean (*N* = 13) waveforms from electrode Cz for unaided and aided conditions (Analog, DigitalA, and DigitalB) with two gain settings (20 and 40 dB) at three input levels (30, 50, and 70 dB SPL).

**Figure 2:**
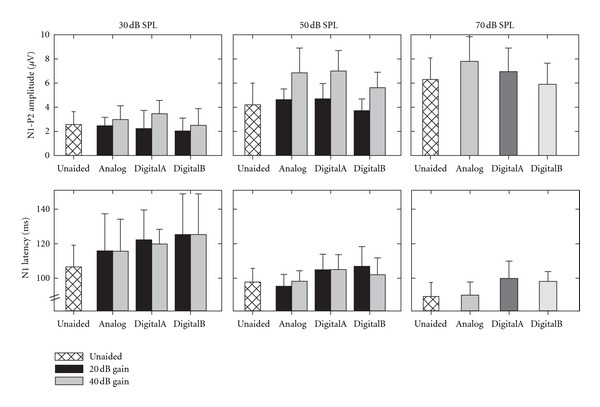
Mean and SD amplitude and latency data for unaided and aided conditions (Analog, DigitalA, and DigitalB) with two gain settings (20 and 40 dB) at three input levels (30, 50 and 70 dB SPL). Sample size is *N* = 13 for all conditions, except for the following latency results: 30 dB SPL/DigitalA/20 dB gain (*N* = 10); 30 dB SPL/DigitalB/20 dB gain (*N* = 11); 30 dB SPL/DigitalB/40 dB gain (*N* = 12).

Grand mean waveforms for unaided compared with 20 and 40 dB of hearing aid gain for three hearing aids (Analog, DigitalA, and Digital B) across three input levels (30, 50, and 70 dB SPL) are shown in Figure 1. Mean (and standard deviation) amplitude and latency results for each condition are presented in Figure 2.

### 3.1. Unaided Compared with 20 dB Gain Condition

In Figures 1 and 2, it is apparent that any differences between unaided and aided response amplitudes in the 20 dB gain condition are quite small, with some aided results even appearing smaller in amplitude than the unaided condition. Indeed, results of the ANOVA revealed a significant interaction between hearing aid type and input level (*F*(6, 72) = 2.48, *ε* = 0.78, *P* = 0.046); Neuman-Keuls post hoc analysis showed that N1-P2 amplitudes in the DigitalB condition were significantly smaller at 50 and 70 dB SPL input levels compared with the other aided conditions as well as the unaided. There were no significant differences between N1-P2 amplitudes at 30 dB SPL with or without hearing aids. At 70 dB SPL, the only hearing aid condition that resulted in larger N1-P2 amplitudes (compared to unaided) was the Analog aid. There were also significant main effects for hearing aid type (*F*(3,36) = 6.99, *ε* = 0.95, *P* < 0.001) and for input level (*F*(2, 24) = 74.97, *ε* = 1.00, *P* < 0.001). These findings indicate that the only 20 dB gain condition in which N1-P2 amplitudes were larger than unaided N1-P2 amplitudes occurred in the analog condition; even this result was not consistent across input levels. Additionally, response amplitudes in the DigitalB condition were significantly smaller than in all other aided and unaided conditions.

Figure 2 shows that N1 latencies in both digital hearing aid conditions appear longer by as much as 10–20 ms, compared with analog and unaided conditions. This was confirmed by ANOVA results, which revealed a significant main effect of hearing aid type (*F*(3, 36) = 15.33, *ε* = 1.00, *P* < 0.001). Post hoc analysis showed that both digital hearing aids resulted in significantly delayed N1 latencies compared with unaided responses. In contrast, there was no significant difference between unaided and aided N1 latencies in Analog hearing aid conditions. There was also a significant main effect of input level (*F*(1, 12) = 47.88, *ε* = 1.00, *P* < 0.001), such that response latencies were longer for the 50 compared with the 70 dB SPL input level. There was a nonsignificant trend (hearing aid type X input level interaction: *F*(3, 36) = 2.42, *ε* = 0.817, *P* = 0.096), such that latencies were the same in unaided and Analog hearing aid conditions for both input levels, but latencies for both digital hearing aids were longer compared with Analog and unaided conditions.

### 3.2. Unaided Compared with 40 dB Gain Condition

In Figures 1 and 2, N1-P2 amplitudes are larger in the 40 dB gain conditions compared with unaided N1-P2 amplitudes at 50 and 70 dB SPL input levels; however, at 30 dB SPL, there appears to be no difference between N1 amplitudes across conditions. Results from the ANOVA revealed a significant main effect of hearing aid type (*F*(3,36) = 7.58, *ε* = 1.00, *P* < 0.001) and input level (*F*(1, 12) = 115.26, *ε* = 1.00, *P* < 0.001), as well as a significant interaction between hearing aid type and input level (*F*(3, 36) = 5.42, *ε* = 0.96, *P* = 0.004). Post hoc analysis showed that N1-P2 amplitudes were significantly smaller in DigitalB aided conditions compared with Analog and DigitalA. Additionally, post hoc analysis of the 40 dB gain results confirmed that N1-P2 amplitudes were larger for all three hearing aids at 50 dB SPL compared with unaided; however, no significant differences existed between N1-P2 amplitudes in aided and unaided conditions at the 30 dB SPL input level, and no significant differences existed between hearing aids at 30 dB SPL. Thus, despite being set with 40 dB of gain, N1-P2 amplitudes were never significantly larger for any of the 30 dB SPL aided conditions compared with unaided at 30 dB SPL.

Similar to findings for the 20 dB gain setting, N1 latencies for the 40 dB gain setting appear longer again, by as much as 10–15 ms, in the digital hearing aid conditions relative to the analog and unaided conditions. This seems to be the case for both 30 and 50 dB SPL input levels; however, as mentioned previously, the latencies for the 30 dB SPL input level were not included in the statistical analysis due to missing data. The ANOVA results revealed a statistically significant difference between hearing aid conditions (*F*(3, 36) = 5.57, *ε* = 1.00, *P* = 0.003). As is evident in Figure 2, there was a trend for both digital hearing aid conditions to be delayed compared with unaided and analog hearing aid conditions; however, N1 latencies were only significantly longer for the DigitalA condition and there was no significant difference between response latencies for the unaided or other hearing aid conditions.

### 3.3. 20 dB Compared with 40 dB Gain Condition

In order to determine whether hearing aid gain had an effect on response amplitudes and/or latencies, results for the two gain settings (20 and 40 dB) were compared across input level (30 and 50 dB SPL for amplitude and 50 dB SPL for latency). It is evident in Figure 2 that N1-P2 amplitudes are larger in the 40 dB compared with the 20 dB gain setting for all hearing aid types. Results from the ANOVA revealed a significant main effect for gain (*F*(1, 12) = 73.18, *ε* = 1.00, *P* < 0.001) and for input level (*F*(1, 12) = 118.13, *ε* = 1.00, *P* < 0.001), as well as a significant interaction between gain setting and input level (*F*(1, 12) = 7.32, *ε* = 1.00, *P* = 0.019). Post hoc analysis showed significantly larger N1-P2 amplitudes for the 40 dB gain setting compared with the 20 dB gain setting at 50 dB SPL; however, no significant difference existed between N1-P2 amplitudes for the two gain settings at the 30 dB SPL input level. The main effect for hearing aid type was also significant (*F*(2, 24) = 6.01, *ε* = 1.00, *P* = 0.008), such that N1-P2 amplitudes were significantly smaller for DigitalB hearing aid condition compared with Analog and DigitalA hearing aid conditions. There was no significant interaction between hearing aid type and gain setting (*F*(2, 24) = 1.02, *ε* = 1.00, *P* = 0.37), hearing aid type and input level (*F*(2, 24) = 1.73, *ε* = 1.00, *P* = 0.20), or hearing aid type by gain setting by input level (*F*(2, 24) = 0.41, *ε* = 1.00, *P* = 0.67). These results are consistent with findings reported previously that there is little to no effect of gain for the 30 dB SPL input level, but a significant effect of gain for the 50 dB SPL input level.

Latency results for the 20 and 40 dB gain settings in Figure 2 show that, once again, there was an obvious difference between N1 latencies across digital and analog aided conditions, such that N1 latencies were longer in the digital hearing aid conditions (particularly for the 20 dB gain setting); however, there did not seem to be an effect of gain on latency for any hearing aid. The ANOVA confirmed a significant main effect for hearing aid type (*F*(2, 24) = 13.66, *ε* = 1.00, *P* < 0.001), such that significantly shorter N1 latencies were obtained in Analog hearing aid conditions compared with either digital hearing aid. No significant main effect of gain setting existed for N1 latencies (*F*(1, 12) = 0.46, *ε* = 1.00, *P* = 0.51), indicating that latencies for the 40 dB gain setting were not significantly shorter than latencies for the 20 dB gain setting. There was a nonsignificant trend for latencies to be shorter in the Analog hearing aid conditions compared with the digital hearing aid conditions across gain settings for the hearing aid type and gain interaction (*F*(2, 24) = 2.92, *ε* = 1.00, *P* = 0.07).

### 3.4. Equivalent Nominal Output Levels

**Figure 3:**
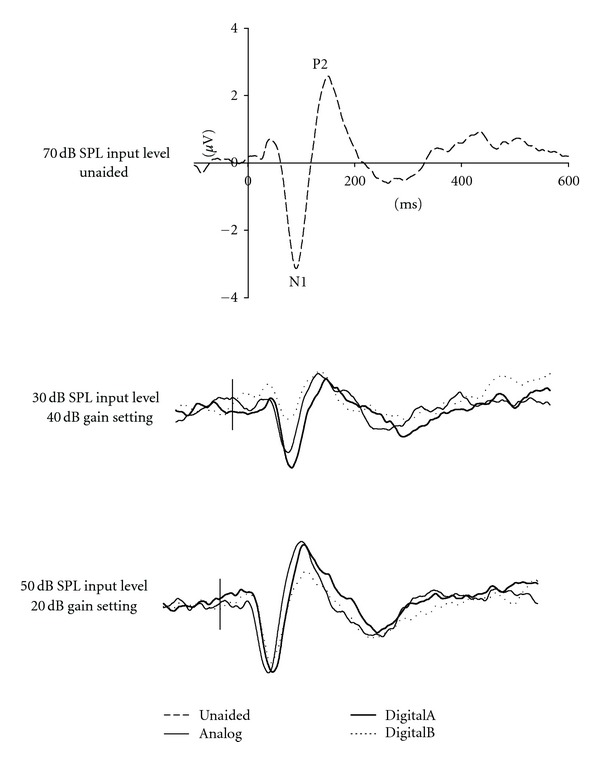
Grand mean (*N* = 13) waveforms for 70 dB SPL equivalent nominal output for unaided and aided conditions (Analog, DigitalA, and DigitalB).

A key question in this study was whether N1-P2 amplitudes would be the same when compared across unaided and aided conditions for the same nominal output level. More specifically, the 70 dB SPL input level for the unaided condition was compared with the 30 dB SPL input level for the 40 dB gain condition and the 50 dB SPL input level for the 20 dB gain condition, because all three combinations would be expected to yield a 70 dB SPL output in the ear canal. Grand mean waveforms for these three combinations of unaided and aided conditions are presented in Figure 3. N1-P2 amplitudes are larger in the unaided compared with the aided conditions, which is confirmed by ANOVA results that revealed a significant main effect across conditions (*F*(6, 72) = 14.75, *ε* = 0.89, *P* < 0.001). Post hoc analysis showed that N1 response amplitudes were significantly smaller for all hearing aid conditions (i.e., 30 dB SPL input level and 40 dB hearing aid gain; 50 dB SPL input level and 20 dB hearing aid gain) compared with the unaided condition (i.e., 70 dB SPL). Although nominal output levels should have been equal, N1-P2 amplitudes obtained with the 50 dB SPL and 20 dB gain conditions were significantly larger than those obtained with the 30 dB SPL and 40 dB gain conditions for all hearing aid conditions except for DigitalB.

## 4. Discussion

The current study, carried out in participants with normal hearing, assessed the effects of processing of stimuli by three different hearing aids and two gain settings on SCP amplitudes and latencies. This differs from many previous studies in participants with hearing loss, where aided SCPs were obtained at levels where unaided stimuli were inaudible and unaided SCPs were absent. Results of the present study clearly demonstrate that, in many instances, introducing hearing aid gain had little or no effect on SCP N1-P2 measures, and, in some comparisons, SCP results worsened (i.e., lower amplitude and/or later latency). Further, significant differences were found between different hearing aids. These findings are despite the fact that verification of REIG measures using conventional hearing aid test procedures in the present study confirmed all three hearing aids provided 20 and 40 dB of gain at input levels used in testing for all subjects.

There has been much interest in using the slow cortical potential as an objective hearing aid measure, especially recently; however, there is clearly conflicting evidence in the literature regarding the accuracy of the SCP for this purpose. Most research indicates that SCPs can indeed be recorded in response to hearing-aid-processed stimuli [24–33]. Studies involving individuals with hearing loss have shown increased SCP response presence, increased SCP amplitudes, and, in some studies, decreased SCP latencies between aided and unaided conditions [27–29, 31–33, 51]. However, these studies did not systematically assess effects of different hearing aid parameters (e.g., gain, frequency response, or hearing aid type) or of input intensity.

Some studies suggest that different cortical responses for different speech stimuli are maintained in aided conditions [27–30]. However, results of several recent studies indicate that the SCP N1-P2 cannot provide a reliable method to objectively demonstrate in individuals that the brain has discriminated between different speech stimuli [51–53]. Because of this, the commercial release of the HEARLab system for recording and analysis of aided and unaided SCPs in infants does not provide for comparisons of waveform shapes in response to different stimuli for the purpose of inferring perception of different (speech) stimuli ([51]; H. Dillon, personal communication).

Several studies in individuals with normal hearing, including the present study, have assessed several aspects of input stimuli and/or hearing aid parameters, including input intensity ((current study; [24, 25]) that gain (current study; [24]), and hearing aid type (current study). All of these studies indicate the aided SCP does not accurately reflect changes in these parameters.

The current study is the first study to assess the effects of amplification by different hearing aids on the SCP. Specifically, the current study investigated SCP responses to stimuli processed by an analog hearing aid, as well as two digital hearing aids from two different manufacturers, all set to provide the same amount of gain. To simplify comparisons, all three hearing aids were set to linear processing, with additional processing features (e.g., feedback reduction; noise reduction) tuned off. As noted previously, despite older technology, the analog hearing aid was included because almost all preceding reports of the SCP in response to hearing-aid-processed stimuli involved participants using analog hearing aids. Importantly, the results of the current study indicate significantly (and clearly) different effects on the SCP for the three different hearing aids, with the DigitalB hearing aid condition showing significantly smaller amplitudes than the other hearing aid conditions, both digital hearing aid conditions showing longer (albeit not quite significant) latencies, and the Analog hearing aid showing larger SCP amplitudes than the digital hearing aid conditions.

Prior to the current study, two studies have reported no significant differences between unaided SCP amplitudes and SCP amplitudes measured while subjects wore hearing aids providing 20 dB of gain [25, 30]. In the current study, none of the hearing aids resulted in significantly larger SCP amplitudes for 30 or 50 dB SPL stimuli and 20 dB gain. Small but significant amplitude increases were seen only for the Analog hearing aid for 70 dB SPL stimuli. Indeed, the DigitalB hearing aid resulted in significantly *smaller* SCP amplitudes (at 50 and 70 dB SPL) compared to unaided results. These results cannot be explained by SCP N1-P2 amplitude reaching a ceiling at higher intensities or the hearing aid reaching its maximum output, because discrepancies were seen at all intensities. Indeed, although previous research indicates increases in SCP amplitude and decreases in latency asymptote at higher intensities, for tonal stimuli these plateaus do not occur until at least 80–90 dB SPL (e.g., [54–58]), although this may differ for individuals with hearing loss [54].

Only two studies, the current study and the recent study by Billings and colleagues [24], have assessed changes in the aided SCP occurring with changes in hearing aid settings using SCPs. Both studies indicate that SCP amplitudes do not accurately reflect the expected hearing gains. In the current study, increasing the gain to 40 dB resulted in significantly larger SCP amplitudes for all three hearing aids, but only at 50 and 70 dB SPL, and not at 30 dB SPL. Importantly, although larger in amplitude for 40 dB gain, the hearing aid conditions resulted in smaller responses compared to the unaided condition at an equivalent nominal output level. Our following study [59] provides some explanations for the results of the current study.

Additionally, N1 latencies were longer in conditions involving digital hearing aids compared with Analog and unaided conditions; there were no significant differences between N1 latencies for unaided and Analog aided conditions. The differences seen, as much as 20 ms, are much longer than the 6.8 ms (DigitalA) and 2.3 ms (DigitalB) hearing aid delays indicated by the standard electroacoustic measures conducted using the Fonix 7000 System. We discuss this further in our following study [59].

Concern has been raised about conclusions drawn from studies in individuals with normal hearing of aided versus unaided SCPs (H. Dillon, personal communication). It is possible that the internal noise of hearing aids could affect SCP results in subjects with normal hearing but would be below threshold in subjects with hearing loss. Alternatively, the hearing aids amplified ambient noise in the sound booth, which could possibly have detrimental effects on SCPs especially in normal listeners. Indeed, recent studies of the SCP indicate large effects of noise on the SCP, such that signal-to-noise ratio (SNR) may be a more determining factor than the absolute stimulus level [24, 36]. We consider SNR in our follow-up study [24]. However, as problems were seen also at the higher input levels (50 and 70 dB SPL), as well as at the lowest level (30 dB SPL), SNR cannot explain all of the current study's results. Further, if studies of aided SCPs are only suited for individuals with hearing loss, it is not clear where the hearing level dividing line should be, given that individuals with regions of mild hearing loss are also fitted with hearing aids. In our view, it is important to better understand how SCP results reflect hearing-aid-processed stimuli in individuals with normal hearing, prior to assessing individuals with hearing loss.

The implications of the current study, as well as other studies, are that SCP results do not always reflect the expected (and electroacoustically verified) gain of hearing aids, and that different hearing aids with similar electroacoustic parameters (i.e., gain) may result in substantially different SCP results. Thus, it would be possible that an appropriately fitted hearing aid (for degree and configuration of the hearing loss) in a client may either show no SCP response or no improvement from the unaided SCP response, even though behaviourally the client shows clear improvement. Similarly, changing hearing aid setting may not change the SCP. Finally, changing the type or brand of hearing aid may result in substantial SCP differences, even though standard electroacoustic measures indicate no change.

## 5. Conclusions

There is much interest in the audiology community in the use of the SCP in the hearing-aid fitting process for infants with hearing loss. The results of the current study (as well as several previous studies) indicate the SCP may not accurately reflect the gain expected from the prescribed hearing aids. Thus, aided-SCP results must be cautiously interpreted when used clinically. A “present” SCP may be interpreted as indicating that the individual's auditory cortex has responded to the hearing-aid-processed stimuli; however, as Dillon and colleagues have recently cautioned, “this does not necessarily indicate that the hearing aids are providing effective amplification” [60]. Importantly, an “absent” SCP in the aided condition does not always indicate that stimuli are inaudible to the individual, as several studies have indicated absent SCPs to audible stimuli as much as 20–30 dB above their behavioural threshold (e.g., [23, 34, 35, 44, 60]).

Clearly, more research concerning the “aided-SCP” technique is required. Importantly, among the needed studies are assessments of the effects of hearing aid processing on the acoustics of the stimuli used to elicit the SCP. Until now, it has been assumed that the longer durations of SCP stimuli allow for adequate processing by hearing aids. SCP stimuli have been contrasted to the much shorter click and brief-tone stimuli required for ABR testing, which were too brief for hearing aid processing and thus did not result in valid measurements of hearing aid gain or output [9–11]. The current study as well as previous studies [24, 25, 30], however, indicate the SCP also does not accurately reflect hearing aid characteristics. In our following study [59], we investigate this further, assessing the acoustic effects of the hearing aid processing on test signals used to elicit the SCP.
